# Different Associations of Plasma Lipopolysaccharide and Lipopolysaccharide-Binding Protein Concentrations with the Deterioration of Energy Metabolism from Healthy Individuals to Patients with Non-Alcoholic Fatty Liver Disease

**DOI:** 10.3390/metabo16020144

**Published:** 2026-02-20

**Authors:** Nobuo Fuke, Yosui Tamaki, Kazunobu Aso, Yu Ota, Shin Otake, Shigenori Suzuki

**Affiliations:** 1 Diet & Well-being Research Institute, KAGOME CO., LTD., 17 Nishitomiyama, Nasushiobara 329-2762, Japan; shigenori_suzuki@kagome.co.jp; 2 Division of Gastroenterology, Department of Internal Medicine, Asahikawa Medical University, Asahikawa 078-8510, Japan; tamaki.yosui.he@mail.hosp.go.jp (Y.T.); y-ota@asahikawa-med.ac.jp (Y.O.); shin-o@asahikawa-med.ac.jp (S.O.); 3 Department of Gastroenterology, National Hospital Organization, Asahikawa Medical Center, Asahikawa 078-8644, Japan

**Keywords:** lipopolysaccharide, lipopolysaccharide-binding protein, metabolic endotoxemia, non-alcoholic fatty liver disease, healthy subjects, cross-sectional study, triglycerides, energy metabolism, lipid metabolism

## Abstract

**Background**: Energy metabolism progressively deteriorates from a healthy state to non-alcoholic fatty liver disease (NAFLD), and circulating lipopolysaccharide (LPS) may contribute to this process. However, previous studies have analyzed healthy individuals and NAFLD patients together, leaving stage-specific associations unclear. Whether LPS and its surrogate marker, lipopolysaccharide-binding protein (LBP), show similar relationships during NAFLD development also remains unknown. This study evaluated the associations between plasma LPS and LBP concentrations with clinical parameters in healthy individuals and NAFLD patients. **Methods**: We conducted a cross-sectional study of 31 healthy individuals (median age [IQR]: 31 (26–43) years) and 31 NAFLD patients (59 (54–70) years). Plasma LPS and LBP concentrations and clinical parameters were measured. Correlations were assessed using Spearman’s rank analysis, followed by multivariate regression adjusting for age, sex, and BMI. **Results**: Plasma LPS and LBP concentrations were significantly higher in NAFLD patients compared to healthy individuals. Additionally, in the univariate regression analysis for all study participants, plasma LPS concentrations were correlated with obesity, blood pressure, liver function, lipid metabolism, and glucose metabolism. Plasma LBP concentrations were also correlated with age, obesity, blood pressure, liver function, lipid metabolism, glucose metabolism, and inflammatory cytokines. In healthy individuals, LPS correlated positively with triglycerides (TG), remaining significant after adjustment and exclusion of participants with any clinical test values outside the normal range. This association was not observed in NAFLD patients. Plasma LBP did not correlate with TG in either group; however, it was inversely associated with hepatic fat fraction in NAFLD patients, although this association was attenuated after adjusting for alanine aminotransferase. **Conclusions**: Plasma LPS correlates with TG even in clinically healthy individuals, suggesting LPS may influence lipid metabolism before NAFLD onset.

## 1. Introduction

Non-alcoholic fatty liver disease (NAFLD) is a chronic liver disease characterized by excessive fat accumulation in the liver among individuals with little to no alcohol consumption. NAFLD is currently the most common chronic liver disease worldwide, affecting an estimated 32.4% of the global adult population [1]. NAFLD encompasses a wide range of conditions, from simple hepatic steatosis to non-alcoholic steatohepatitis (NASH), and can progress to liver fibrosis, cirrhosis, and even hepatocellular carcinoma. The rising prevalence of NAFLD is closely linked to the global epidemics of obesity, type 2 diabetes, and metabolic syndrome, representing a significant public health challenge with considerable economic burdens on healthcare systems. Therefore, it is essential to elucidate the factors contributing to the onset of NAFLD and establish prevention strategies.

Epidemiological studies conducted in Japan have shown that gradual weight gain begins approximately five years prior to the onset of NAFLD [2]. Furthermore, a study tracking 2666 individuals without NAFLD over 16 years reported that changes in waist circumference are associated with the development of NAFLD [3]. These previous reports suggest that a gradual deterioration in energy metabolism occurs as individuals progress from a healthy state to the onset of NAFLD.

Such metabolic deterioration is characterized by coordinated abnormalities in glucose and lipid metabolism across multiple organs, as summarized in previous reviews [4]. In brief, overnutrition and physical inactivity promote insulin resistance in skeletal muscle [5], reducing glucose uptake and increasing the flux of glucose to the liver. In hepatocytes, excess glucose is redirected toward de novo lipogenesis, while insulin resistance in adipose tissue enhances the release of free fatty acids and glycerol into the circulation. The increased hepatic influx of these substrates accelerates triglyceride synthesis, promotes very-low-density lipoprotein (VLDL) secretion, and elevates plasma triglyceride levels [6]. As NAFLD develops, these changes are accompanied by further dysregulation of mitochondrial β-oxidation and gluconeogenesis, thereby reinforcing abnormalities in both lipid and glucose metabolism.

One of the factors thought to contribute to the deterioration of energy metabolism is lipopolysaccharide (LPS). LPS is a molecule found in the outer membrane of Gram-negative bacteria, consisting of lipids and polysaccharides. LPS, known as an endotoxin, activates Toll-like receptor 4 (TLR4), triggering inflammatory responses in humans and animals. Previously, it was believed that LPS only enters human blood during pathological conditions such as infections or colitis. However, a study by Cani et al. suggested that a high-fat diet increases the influx of intestinal-derived LPS, causing inflammation in the liver and adipose tissue, leading to abnormalities in glucose metabolism and obesity, thereby resulting in a fatty liver-like condition [7]. In light of this, epidemiological studies focusing on the association between NAFLD and blood LPS concentrations have been conducted. These studies use both LPS itself and LPS-binding protein (LBP) as indicators and investigate their associations with clinical parameters. LBP is a protein mainly produced in the liver in response to LPS [8]. Therefore, in human studies, blood LBP is often used as an alternative marker of circulating LPS exposure [9,10,11]. Past epidemiological studies have reported associations between both blood LPS and blood LBP with lipid metabolism, glucose metabolism, liver function, and hepatic fat. Specifically, in a case–control study by Wong et al., an analysis including both healthy individuals and NAFLD patients found that blood LPS concentrations were positively correlated with aspartate aminotransferase (AST), homeostasis model assessment of insulin resistance (HOMA-IR), total cholesterol (TC), low-density lipoprotein cholesterol (LDL-c), and triglycerides (TG), and negatively correlated with high-density lipoprotein cholesterol (HDL-c) [12]. Blood LBP concentrations were positively correlated with keratin 18 fragments (a marker for NASH), alanine aminotransferase (ALT), AST, fasting blood glucose (FBG), HOMA-IR, TC, LDL-c, TG, hepatic TG, and future NAFLD risk, while negatively correlated with HDL-c. In a case–control study by du Plessis et al., an analysis of healthy individuals and NAFLD patients showed positive correlations between blood LPS concentrations, body fat percentage, C-reactive protein, and ALT [13]. These reports, similar to those in mice [7], suggest that blood LPS may play a role in the development of NAFLD in humans. However, these epidemiological studies conducted statistical analyses including both healthy individuals and NAFLD patients. Therefore, it remains unclear how blood LPS is associated with stage-specific health conditions during the gradual deterioration of energy metabolism from a healthy state to the onset and progression of NAFLD. Furthermore, previous epidemiological studies have used both LPS and LBP as indicators. As mentioned earlier, LBP is a protein produced in the liver in response to LPS, but it has also been reported to be secreted independently of LPS [14]. Therefore, it is unclear whether blood LPS and blood LBP show the same associations with health conditions during the gradual deterioration of energy metabolism from a healthy state to the development of NAFLD.

In this study, we evaluated the associations between various clinical parameters and plasma LPS and LBP concentrations in both healthy individuals and NAFLD patients. Specifically, we first assessed the associations between plasma LPS or plasma LBP concentrations and each clinical parameter using simple correlation analysis for the entire study population, for healthy individuals only, and for NAFLD patients only. Then, for the parameters showing significant associations in the simple correlation analysis, we performed multiple regression analyses to confirm the robustness of these associations. In conclusion, plasma LPS correlates with TG even in clinically healthy individuals, suggesting LPS may influence lipid metabolism before NAFLD onset.

## 2. Materials and Methods

### 2.1. Ethical Approval and Consent to Participate

This study was conducted in accordance with the Declaration of Helsinki (2013), and all procedures involving human subjects were approved by the Asahikawa Medical University Research Ethics Committee (Approval No. 18091) and the KAGOME CO., LTD. Research Ethics Review Committee (Approval No. 2018-R04). Written informed consent was obtained from all study participants.

### 2.2. Study Design

This was a cross-sectional study conducted in Asahikawa City, Hokkaido, Japan, from September 2018 to December 2019.

### 2.3. Subjects

In this study, healthy individuals and NAFLD patients were selected as study participants. The inclusion criteria for each group are described below. The nomenclature and diagnostic criteria for fatty liver disease were revised in 2023, and the category of NAFLD was officially replaced by metabolic dysfunction–associated steatotic liver disease (MASLD). However, this study was conducted between September 2018 and December 2019, prior to the revision of these definitions. Eligibility was determined according to the diagnostic criteria for NAFLD that were in use at that time. Therefore, throughout this manuscript, we refer to the condition as NAFLD to ensure consistency with the original study protocol and data interpretation.

#### 2.3.1. Healthy Individuals

Since NAFLD is associated with metabolic syndrome [15], individuals with all metabolic syndrome-related parameters within normal ranges were classified as healthy individuals. Specifically, the study participants were men and women aged 20 years and older from Asahikawa Medical University and Asahikawa Medical University Hospital who met the following criteria based on a health checkup conducted within the past three months: (1) Waist circumference: <85 cm for men and <90 cm for women, (2) Fasting blood glucose (FBG) <110 mg/dL, (3) Triglycerides (TG) <150 mg/dL, (4) High-density lipoprotein cholesterol (HDL-c) ≥40 mg/dL, (5) Systolic blood pressure (SBP) <130 mmHg and diastolic blood pressure (DBP) <85 mmHg.

#### 2.3.2. NAFLD Patients

Previous reports have indicated that NAFLD does not improve significantly if weight loss is less than 7% [16]. Therefore, the inclusion criteria for NAFLD patients were men and women aged 20 years and older who were attending the Diabetes Department and Gastroenterology Department at Asahikawa Medical University Hospital, had been previously diagnosed with fatty liver by ultrasonography, and had experienced less than a 7% weight loss since that time.

The exclusion criteria for both groups were as follows: (1) Individuals suspected of having liver diseases other than NAFLD, specifically: (a) Ethanol consumption exceeding 30 g/day for men and 20 g/day for women, (b) Hepatitis B or C, (c) Drug-induced liver injury, (d) Autoimmune hepatitis, (e) Primary biliary cirrhosis, (f) Wilson’s disease, (g) Hemochromatosis, and h) Citrin deficiency. (2) Individuals with factors that could alter blood LPS concentrations other than NAFLD, specifically: (a) Individuals with gastrointestinal diseases, (b) Individuals who have taken antibiotics within one month prior to blood collection, (c) Individuals who have had infections within one month prior to blood collection. (3) Individuals with personal backgrounds that could lead to identification, (4) Individuals for whom data collection planned for this study would be difficult, (5) Any other individuals deemed inappropriate by the principal investigator.

### 2.4. Schedule

#### 2.4.1. Healthy Individuals

Participants were recruited via email and poster announcements during the health checkup period. For those who provided consent after the study plan was explained, a questionnaire and blood collection were conducted on the same day, within three months of receiving the health checkup results. Participants were instructed to fast from 9:00 PM the day before blood collection until the time of blood sampling.

#### 2.4.2. NAFLD Patients

For NAFLD patients attending regular clinic visits, the study details were explained during the routine consultation. After consent was obtained, data collection was conducted on the same day as the next regular consultation. Participants were instructed to fast from 9:00 PM the day before blood collection until the time of blood sampling.

### 2.5. Data Collection

#### 2.5.1. Medical Interview

##### Healthy Individuals

A questionnaire completed by the study participants was used as the basis for the interview. The questionnaire included the following items: sex, age, body mass index (BMI), waist circumference, blood pressure, blood biochemical test values (ALT, AST, gamma-glutamyl transferase [γ-GT], TG, TC, HDL-c, LDL-c, FBG, Hemoglobin A1c [HbA1c]), medical history, current illness, medication use, supplement use, smoking habits, and drinking habits. BMI, waist circumference, blood pressure, and blood biochemical test values were recorded based on health checkup results obtained within the past three months. In this study, the insulin resistance index HOMA-IR, calculated from FBG and insulin concentrations, was used in the analysis; however, insulin concentrations are not typically measured in regular health checkups. Therefore, the FBG recorded in the questionnaire was used solely to confirm eligibility, and FBG for the calculation of HOMA-IR was measured, along with insulin.

##### NAFLD Patients

The interview was conducted based on the questionnaire completed by the study participants. The questionnaire included the following items: sex, age, medical history, current illness, medication use, supplement use, smoking habits, and drinking habits.

#### 2.5.2. Blood Samples

Blood collection was performed using vacuum blood collection tubes, with 5 mL collected from healthy individuals and 15 mL from NAFLD patients. The samples were then centrifuged at 1200× *g* at 4 °C for 15 min to obtain heparin plasma. The plasma collected was immediately used for analysis on the same day. In healthy individuals, the measurements of LPS concentration, FBG, and insulin concentrations were performed. In NAFLD patients, LPS concentration was measured, and blood biochemical tests (platelet count, ALT, AST, γ-GT, TG, TC, HDL-c, LDL-c, ferritin, hyaluronic acid, 7S domain of type IV collagen, Mac-2 Binding Protein Glycosylation Isomer [M2BPGi], FBG, insulin, HbA1c) were conducted. Serum ferritin was analyzed as a clinical indicator associated with NAFLD severity [17] rather than as a mechanistic marker of inflammation or iron overload. Additionally, some plasma samples from both groups were frozen for later analysis of tumor necrosis factor-alpha (TNF-α), interleukin-6 (IL-6), transforming growth factor-beta 1 (TGF-β1), and LBP concentrations. TNF-α, IL-6, and TGF-β1 are proteins induced by LPS and have been reported in in vitro studies to potentially contribute to liver fibrosis [18], which is why they were measured in this study.

#### 2.5.3. Analysis of Plasma LPS Concentrations

The measurement of plasma LPS concentration was performed using the method previously reported [19]. Specifically, plasma samples were diluted 10-fold with LPS-free distilled water (Otsuka Pharmaceutical Co., Ltd., Tokyo, Japan) and heated at 70 °C for 10 min. Then, 50 μL of each sample was mixed with limulus amoebocyte lysate (LAL) reagent (Endospecy; Seikagaku Corporation, Tokyo, Japan) on a 96-well plate (AGC Techno Glass Co., Ltd., Shizuoka, Japan), and incubated at 37 °C for 60 min (Plate 1). To correct for absorbance (Abs) due to plasma color, 50 μL of plasma was mixed with LPS-free distilled water on a 96-well plate and incubated at 37 °C for 60 min (Plate 2). After incubation, Abs at 405 nm (with a reference wavelength at 492 nm) was measured using a microplate reader (CORONA ELECTRIC Co. Ltd., Ibaraki, Japan). The Abs due to the LAL reaction were then calculated as follows:

(Abs of ‘plasma + LAL reagent’ well [plate 1]) − (Abs of blank well [plate 1]) − ([Abs of ‘plasma + water’ well {plate 2}] − [Abs of blank well {plate 2}])

Control standard endotoxin (Seikagaku Corporation) ranging from 0.0001 to 0.1000 EU/mL was reacted with the LAL reagent in the same manner as the plasma samples on Plate 1. The LPS concentration in the plasma samples was calculated from a standard curve prepared using the control standard endotoxin. If the plasma LPS concentration was below the detection limit (0.0001 EU/mL), it was set to 0 EU/mL.

#### 2.5.4. Physiological and Biochemical Analyses

Weight, height, waist circumference, blood pressure, and blood biochemical tests (platelet count, ALT, AST, γ-GT, TG, TC, HDL-c, LDL-c, ferritin, hyaluronic acid, 7S domain of type IV collagen, M2BPGi, FBG, insulin and HbA1c) were measured following the standard clinical testing methods at Asahikawa Medical University Hospital. The degree of liver fibrosis and hepatic steatosis was assessed using magnetic resonance elastography (MRE) and magnetic resonance imaging proton density fat fraction (MRI-PDFF), respectively, in accordance with the standard clinical testing methods at Asahikawa Medical University Hospital. The classification methods for liver fibrosis stage and hepatic steatosis grade were as follows: Liver fibrosis stage: F0, <2.61 kPa (no fibrosis), F1–2, 2.62–3.61 kPa (mild), F3–4, ≥3.62 kPa (advanced); Hepatic steatosis grade: G1, <11.3%, G2, 11.3–17.0%, G3, ≥17.1%. The Fibrosis-4 (FIB-4) index was calculated according to the following formula [20]:

[Age (years) × AST (U/L)]/[Platelet count (10^9^/L) × √ALT (U/L)]

The measurements of TNF-α (TNF-α Human ELISA Kit Quantikine HS 5th Generation, R&D Systems, Minneapolis, MN, USA), IL-6 (IL-6 Human ELISA Kit Quantikine HS 2nd Generation, R&D Systems), TGF-β1 (TGF-β1 Human DuoSet Kit, R&D Systems), and LBP (Human LBP DuoSet, R&D Systems) were performed following the protocols provided with each respective measurement kit.

### 2.6. Statistical Methods

The sample size for this study was calculated based on a previous report that evaluated the correlation between blood LPS concentrations and clinical parameters in diabetic patients [21]. Assuming a correlation coefficient of 0.477 (the highest correlation coefficient reported in the paper), and using a significance level of 5% (two-sided) and a power of 80%, the required sample size was estimated. As a result, the sample size needed for this study was estimated to be ≥29 per group.

Data are presented as the median and interquartile range (IQR), unless otherwise noted. Since many clinical parameters were presumed not to follow a normal distribution based on previous reports [22], comparisons between two groups for continuous variables were performed using the Mann–Whitney *U* test. When comparing continuous variables across three or more groups, the Kruskal–Wallis test was used, and if a significant result was obtained, the Steel test was performed. Comparison of proportions between two groups was done using Fisher’s exact test. For univariate correlation analysis of plasma LPS, LBP, TNF-α, or IL-6 concentrations and clinical parameters, Spearman’s rank correlation coefficient was calculated.

For clinical parameters that showed significant associations with plasma LPS or LBP concentrations in univariate regression analyses or the Mann–Whitney *U* test, multivariate regression or logistic regression analyses were performed to validate the associations. For clinical parameters associated with plasma LPS concentrations, multivariate regression or logistic regression analysis was conducted with each clinical parameter as the dependent variable, plasma LPS concentration as the independent variable, and age, sex, BMI, as well as plasma LBP concentration, plasma TNF-α concentration, plasma IL-6 concentration, plasma TGF-β1 concentration, or the use of angiotensin II receptor blockers (ARB) or calcium channel blockers (CCB) as covariates. In the analysis of the association between plasma LPS concentration and age, plasma LPS concentration was set as the dependent variable, age as the independent variable, and sex, BMI, M2BPGi, hyaluronic acid, or the use of ARB or CCB as covariates in multivariate regression analysis. In the analysis of the association between plasma LPS concentration and the use of ARB or CCB, plasma LPS concentration was the dependent variable, the use of ARB or CCB was the independent variable, and sex and BMI, along with blood pressure (SBP, DBP), lipid metabolism-related indices (LDL-c, HDL-c, TG, TC), or liver function indices (ALT, AST, γ-GT) were included as covariates in multivariate regression analysis. For clinical parameters associated with plasma LBP concentrations, multivariate regression analysis was performed with each clinical parameter as the dependent variable, plasma LBP concentration as the independent variable, and age, sex, and BMI, along with AST, ALT, or γ-GT as covariates. Variables for multivariate regression and logistic regression analyses were subjected to the Shapiro–Wilk normality test, and variables that did not follow a normal distribution were subsequently log-transformed. Since plasma LPS concentrations include 0, 1 was added to all sample values before log transformation [22]. For the important results indicating the relationship between plasma LPS concentration and energy metabolism, QQ plots were generated, and the normality of residuals was checked.

In healthy individuals, an analysis was also performed for participants whose clinical test values were within the reference ranges. The reference ranges for BMI, waist circumference, SBP, DBP, AST, ALT, γ-GT, TG, HDL-c, LDL-c, FBG, and HbA1c were based on the criteria set by the Japan Society of Ningen Dock and Preventive Medical Care [23]. For insulin and HOMA-IR, the reference values set by the Japan Diabetes Society were used [24]. The reference values for each clinical test are as follows: BMI; 18.5–24.9 kg/m^2^, waist circumference; ≤84.9 cm for men, ≤89.9 cm for women, SBP; ≤129 mmHg, DBP; ≤84 mmHg, AST; ≤30 U/L, ALT; ≤30 U/L, γ-GT; ≤50 U/L, TG; 30–149 mg/dL, HDL-c; ≥40 mg/dL, LDL-c; 60–119 mg/dL, FBG; ≤99 mg/dL, HbA1c; ≤5.5%, insulin; ≤15 μIU/mL, HOMA-IR; ≤1.6.

To evaluate the ability of plasma LPS and LBP concentrations to identify NAFLD, receiver operating characteristic (ROC) curves were plotted, and the area under the curve (AUC), as well as sensitivity and specificity for various cut-off values, were calculated.

**Figure 1:**
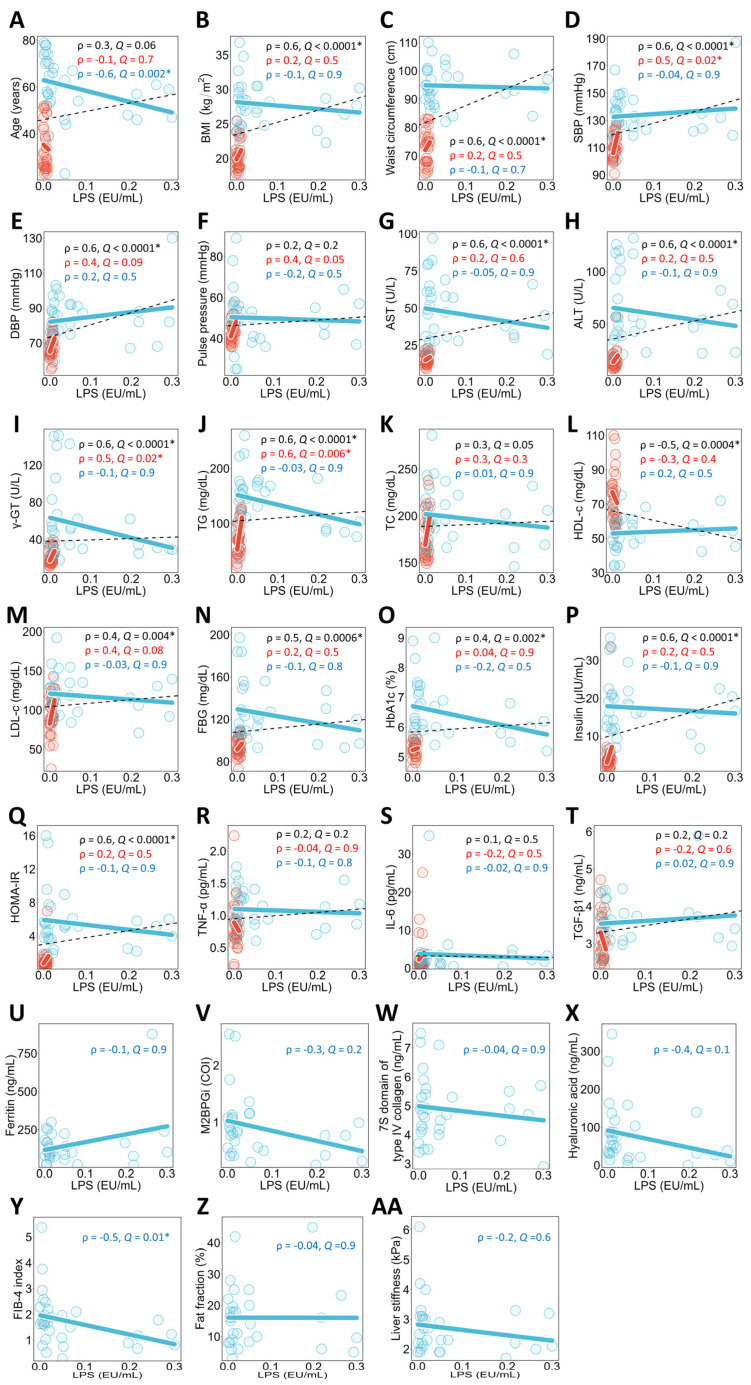
Univariate correlation analysis between plasma LPS concentrations and clinical parameters. Spearman’s rank correlation analysis was performed in all study participants, healthy individuals, and NAFLD patients. Sub-figures are grouped by clinical categories: (**A**) age; (**B**,**C**) obesity-related indices; (**D**,**E**) blood pressure; (**F**) pulse pressure; (**G**–**I**) liver function markers; (**J**–**M**) lipid metabolism markers; (**N**–**Q**) glucose metabolism markers; (**R**–**T**) inflammatory markers; (**U**–**AA**) NAFLD-related markers. The black dashed line in the graph represents the regression line for the correlation between plasma LPS concentrations and each clinical parameter in all study participants, the red solid line in healthy individuals, and the blue solid line in NAFLD patients. The red and blue circles represent the individual values of healthy individuals and NAFLD patients, respectively. The black, red, and blue text in the graph show the Spearman’s rank correlation coefficient (ρ) and *Q*-value for all study participants, healthy individuals, and NAFLD patients. The sample sizes for all study participants, healthy individuals, and NAFLD patients were n = 62, n = 31, and n = 31, respectively. * *Q* < 0.05. NAFLD, non-alcoholic fatty liver disease; BMI, body mass index; SBP, systolic blood pressure; DBP, diastolic blood pressure; AST, aspartate aminotransferase; ALT, alanine aminotransferase; γ-GT, γ-glutamyltransferase; TG, triglycerides; TC, total cholesterol; HDL-c, high-density lipoprotein cholesterol; LDL-c, low-density lipoprotein cholesterol; FBG, fasting blood glucose; HbA1c, hemoglobin A1c; HOMA-IR, homeostasis model assessment of insulin resistance; TNF-α, tumor necrosis factor-α; IL-6, interleukin-6; TGF-β1, transforming growth factor-β1; LPS, lipopolysaccharide; M2BPGi, Mac2 binding protein glycosylation isomer; COI, cut-off index; FIB-4 index, fibrosis-4 index.

In this study, when analyzing the associations between plasma LPS or LBP concentrations and clinical parameters or medication use, the same statistical analyses were repeated for the same objectives (Figures 1, 3, 6 and 8). Therefore, the *p*-values for these analyses were adjusted using the Benjamini–Hochberg method separately within each figure. Generally, the threshold for the false discovery rate (FDR) is chosen between 0.05 to detect strong results [25], 0.1 to detect promising results [25], and 0.2 to detect potential results while considering the possibility of false positives [26]. Since the above analysis serves as an important screening to identify items associated with plasma LPS or LBP concentrations, the FDR threshold was set at *Q* = 0.05.

The specific statistical methods used are listed in the footnotes of the tables and figures. Statistical analyses were performed using JMP^®^ 14 (SAS Institute Inc., Cary, NC, USA) or R version 4.3.1. All statistical analyses were conducted using two-sided tests, with a significance level set at *p* < 0.05 or *Q* < 0.05. Furthermore, results with 0.05 ≤ *p* < 0.1 or 0.05 ≤ *Q* < 0.1 were considered as showing a trend.

## 3. Results

### 3.1. Characteristics of the Study Participants

A total of 31 healthy individuals and 31 NAFLD patients participated in this study. No significant differences were observed between the groups in the sex ratio, the proportion of habitual alcohol drinkers, or the proportion of habitual smokers (Table 1). In contrast, the age of NAFLD patients was significantly higher than that of healthy individuals. Obesity indices (BMI, waist circumference), blood pressure (SBP, DBP), arterial stiffness indices (pulse pressure), liver function indices (AST, ALT, γ-GT), lipid metabolism indices (TG, TC, HDL-c, LDL-c), and glucose metabolism indices (FBG, HbA1c, insulin, HOMA-IR) all showed significantly less favorable profiles in NAFLD patients compared to healthy individuals. Additionally, inflammatory markers (TNF-α, IL-6, TGF-β1), plasma LPS concentrations, and plasma LBP concentrations were significantly higher in NAFLD patients than in healthy individuals. For data listed below ferritin in Table 1, since these were collected only from NAFLD patients, no comparisons with healthy individuals were made.

To explore whether inflammatory activity was associated with metabolic dysfunction in this cohort, correlations between inflammatory cytokines (TNF-α and IL-6) and metabolic parameters were examined. TGF-β1 was excluded from the analysis because it primarily functions as an anti-inflammatory cytokine and is mainly involved in fibrotic processes, particularly through its interaction with IL-6 [27], which is distinct from the inflammatory mechanisms related to metabolic dysfunction examined in this study. Similar to the analyses shown in Figure 1, comprehensive univariate correlation analyses (Spearman’s rank correlation) were conducted between TNF-α or IL-6 and other clinical parameters. In healthy individuals, no significant correlations were observed. In contrast, in NAFLD patients, TNF-α showed significant positive correlations with the FIB-4 index (ρ = 0.57, Q < 0.05), M2BPGi (ρ = 0.52, Q < 0.05), and age (ρ = 0.52, Q < 0.05). In addition, IL-6 was positively correlated with systolic blood pressure (ρ = 0.55, Q < 0.05). These findings indicate that associations between inflammatory activity and metabolic abnormalities were partially present in NAFLD patients in this cohort.

### 3.2. Associations Between Plasma LPS Concentrations and Clinical Parameters in All Study Participants, Healthy Individuals, and NAFLD Patients

A univariate correlation analysis between plasma LPS concentrations and each clinical parameter was conducted for all study participants (Figure 1). The results showed that plasma LPS concentrations were significantly positively correlated with BMI (Figure 1B), waist circumference (Figure 1C), SBP (Figure 1D), DBP (Figure 1E), AST (Figure 1G), ALT (Figure 1H), γ-GT (Figure 1I), TG (Figure 1J), LDL-c (Figure 1M), FBG (Figure 1N), HbA1c (Figure 1O), insulin (Figure 1P), and HOMA-IR (Figure 1Q), and showed trends toward a positive correlation with age (Figure 1A) and TC (Figure 1K). Additionally, plasma LPS concentrations were significantly negatively correlated with HDL-c (Figure 1L).

These univariate correlation analyses between plasma LPS concentrations and clinical parameters were conducted within each group of healthy individuals and NAFLD patients. The results showed that in healthy individuals, plasma LPS concentrations were significantly positively correlated with SBP (Figure 1D), γ-GT (Figure 1I), and TG (Figure 1J), and showed a trend toward a positive correlation with DBP (Figure 1E), pulse pressure (Figure 1F), and LDL-c (Figure 1M). In NAFLD patients, plasma LPS concentrations showed a significant negative correlation with age (Figure 1A) and FIB-4 (Figure 1Y).

**Figure 2:**
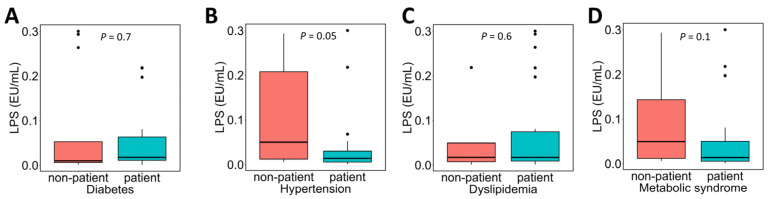
Association between complications and plasma LPS concentrations in NAFLD patients. NAFLD patients were divided into two groups based on the presence or absence of each complication, and a comparison of plasma LPS concentrations between the groups was performed. Subfigures show comparisons according to the presence or absence of the following conditions: (**A**) diabetes, (**B**) hypertension, (**C**) dyslipidemia, and (**D**) metabolic syndrome. In the box plot, the horizontal lines represent the first quartile, median, and third quartile, and the ends of the whiskers represent the minimum and maximum values. Dots above the maximum value indicate outliers. The sample sizes (n) for each comparison based on the presence or absence of each condition (yes:no) were as follows: diabetes, 18:13; hypertension, 20:11; dyslipidemia, 23:18; and metabolic syndrome, 20:11. The Mann–Whitney *U* test was used. LPS, lipopolysaccharide; NAFLD, non-alcoholic fatty liver disease.

The association between the presence or absence of various complications in NAFLD patients and plasma LPS concentrations was examined (Figure 2). Plasma LPS concentrations tended to be lower in patients with hypertension (Figure 2B; patients without hypertension 0.05 [0.01–0.21] EU/mL vs. patients with hypertension 0.01 [0.006–0.03] EU/mL), but no significant differences were observed in comparisons based on the presence or absence of other complications.

**Figure 3:**
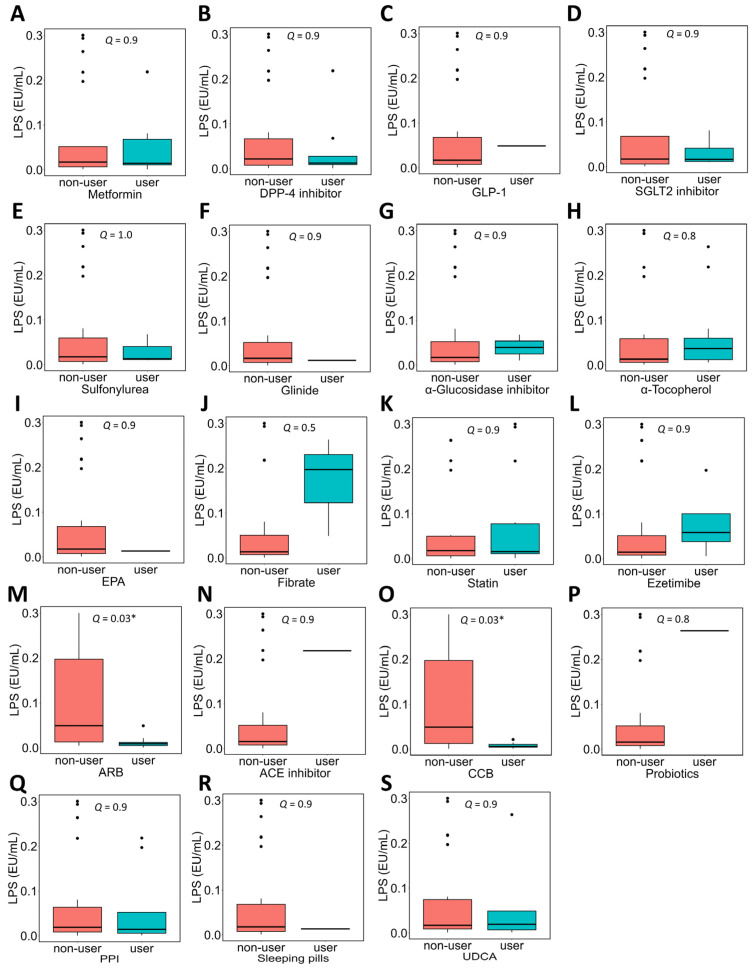
Association between medication use and plasma LPS concentrations in NAFLD patients. NAFLD patients were divided into two groups based on the use of each medication, and a comparison of plasma LPS concentrations between the groups was performed. Subfigures (**A**–**S**) show comparisons of plasma LPS concentrations according to the use or non-use of each medication. In the box plot, the horizontal lines represent the first quartile, median, and third quartile, and the ends of the whiskers represent the minimum and maximum values. Dots above or below the minimum value indicate outliers. The sample sizes (n) for each medication, defined by use or non-use (yes:no), were as follows: metformin, 9:22; DPP-4 inhibitor, 8:23; GLP-1, 1:30; SGLT2 inhibitor, 6:25; sulfonylurea, 3:28; glinide, 1:30; α-glucosidase inhibitor, 2:29; α-tocopherol, 12:19; EPA, 2:29; fibrate, 3:28; statin, 14:17; ezetimibe, 4:27; ARB, 10:21; ACE inhibitor, 1:30; CCB, 9:21; probiotics, 1:30; PPI, 9:22; sleeping pills, 2:29; and UDCA, 8:23. Mann–Whitney *U* test was used. * *Q* < 0.05. LPS, lipopolysaccharide; NAFLD, non-alcoholic fatty liver disease; DPP4, dipeptidyl peptidase 4; GLP-1, glucagon-like peptide-1; SGLT2, sodium glucose cotransporter 2; EPA, eicosapentaenoic acid; ARB, angiotensin II receptor blocker; ACE, angiotensin-converting enzyme; CCB, calcium channel blocker; PPI, proton pump inhibitor; UDCA, ursodeoxycholic acid.

In NAFLD patients, the association between the use of each medication and plasma LPS concentrations was evaluated (Figure 3). Patients taking ARBs (Figure 3M) or CCBs (Figure 3O) showed significantly lower plasma LPS concentrations than those not taking these medications.

**Figure 4:**
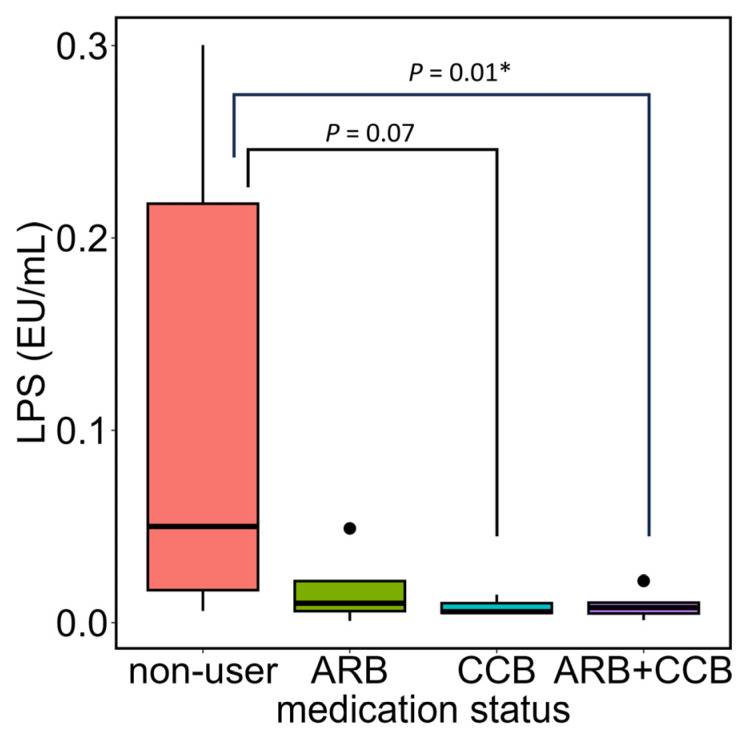
Association between antihypertensive medication use patterns and plasma LPS concentrations. NAFLD patients were divided into four groups based on their use of ARB and CCB, and a comparison of plasma LPS concentrations between the groups was performed. In the box plot, the horizontal lines represent the first quartile, median, and third quartile from the bottom, and the ends of the whiskers represent the minimum and maximum values. Dots above the maximum value indicate outliers. The groups of individuals not taking either ARB or CCB, taking only ARB, taking only CCB, and taking both ARB and CCB included n = 17, n = 4, n = 3, and n = 6, respectively. A Kruskal–Wallis test was first performed, and if significant differences were found, a Steel test was conducted using individuals not taking any medication as the control. * *p* < 0.05. LPS, lipopolysaccharide; NAFLD, non-alcoholic fatty liver disease; ARB, angiotensin II receptor blocker; CCB, calcium channel blocker.

Regarding the use of ARB or CCB, there were 17 individuals who were not taking either medication, 4 individuals taking only ARB, and 3 individuals taking only CCB, while 6 individuals were taking both ARB and CCB; Accordingly, individuals not taking any medication were used as the control group, and plasma LPS concentrations were compared between the groups taking only ARB, only CCB, and both ARB and CCB (Figure 4). A Kruskal–Wallis test was used to compare plasma LPS concentrations across the four groups, and the result was *p* = 0.005. As a post hoc test, a Steel test was performed with individuals not taking any medication as the control group. The results showed that, compared to those not taking any medication, there was no statistically significant difference in plasma LPS concentrations in the ARB-only group (*p* = 0.1), but there was a trend toward lower levels in the CCB-only group (*p* = 0.07), whereas the combination of ARB and CCB showed significantly lower plasma LPS concentrations (*p* = 0.01).

### 3.3. Multivariate Regression Analysis Between Plasma LPS Concentrations and Clinical Parameters in Healthy Individuals

For the clinical parameters that showed a univariate correlation with plasma LPS concentrations in healthy individuals, multivariate regression analyses were performed with each clinical parameter as the dependent variable, plasma LPS concentration as the independent variable, and age, sex, and BMI as covariates. In performing the multivariate regression analysis, the normality of each variable was checked using the Shapiro–Wilk test. Accordingly, plasma LPS concentration (*p* = 0.005), age (*p* = 0.001), BMI (*p* = 0.03), and γ-GT (*p* = 0.004) were confirmed to be non-normally distributed, so their log-transformed values were used in the multivariate regression analysis. The results showed that TG had a significant positive correlation with plasma LPS concentrations, while SBP and γ-GT showed a positive correlation trend (Table 2; Model 2).

In healthy individuals, according to the blood biochemical tests and questionnaire conducted for this study, several participants were found to have clinical test values outside the reference range. Specifically, 7 cases for fasting blood glucose (102–109 mg/dL), 1 case for insulin (33.6 μIU/mL), 3 cases for HOMA-IR (1.6–7.0), 1 case for BMI (25.4 kg/m^2^), 1 case for ALT (57 U/L), 4 cases for LDL (124–143 mg/dL), and 4 cases for HbA1c (5.6–5.7%). These cases had overlaps, and a total of 14 participants had at least one value outside the reference range. After excluding these participants, multivariate regression analysis of plasma LPS concentrations and each clinical parameter with significant correlations in Figure 1 was performed again using only the participants without abnormal clinical test values. First, the normality of each variable was confirmed using the Shapiro–Wilk test, and accordingly, plasma LPS concentration (*p* = 0.003) and γ-GT (*p* = 0.02) were confirmed to be non-normally distributed, so their log-transformed values were used in the multivariate regression analysis. The results showed that even in healthy individuals with no values outside the reference range, a significant positive correlation was found between TG and plasma LPS concentration (Table 3, Model 2).

Furthermore, for TG, multivariate regression analysis was performed in healthy individuals with no abnormal clinical test values, with TG as the dependent variable, plasma LPS concentration as the independent variable, and age, sex, BMI, as well as plasma LBP concentration, TNF-α, IL-6, or TGF-β1 as covariates. The normality of plasma LBP concentration, TNF-α, IL-6, and TGF-β1 was confirmed using the Shapiro–Wilk test, and as a result, plasma LBP concentration (*p* = 0.002), IL-6 (*p* = 3 × 10^−6^), and TGF-β1 (*p* = 0.02) were confirmed to be non-normally distributed, so their log-transformed values were used in the multivariate regression analysis. However, in all analyses, a significant or borderline trend-level correlation between TG and plasma LPS concentration was maintained (*p* = 0.04, 0.05, 0.05, 0.04, respectively).

### 3.4. Multivariate Regression Analysis of Plasma LPS Concentrations and Clinical Parameters in NAFLD Patients

For the FIB-4 index, which showed an association with plasma LPS concentrations in NAFLD patients, multivariate regression analyses were performed with the FIB-4 index as the dependent variable, plasma LPS concentration as the independent variable, and age, sex, and BMI as covariates. In performing the multivariate regression analysis, the normality of each variable was checked using the Shapiro–Wilk test. Accordingly, plasma LPS concentration (*p* = 5 × 10^−7^) and the FIB-4 index (*p* = 0.001) were confirmed to be non-normally distributed, so their log-transformed values were used in the multivariate regression analysis. The results showed that the significant association between the FIB-4 index and plasma LPS concentration was no longer statistically significant (Table 4, Model 2). A multivariate regression analysis was also performed with the FIB-4 index as the dependent variable, LPS as the independent variable, and age, sex, BMI, and the use of ARB or CCB as covariates. However, even in this model, no significant association between plasma LPS concentration and the FIB-4 index was observed (Table 4, Model 3).

In the NAFLD group, the factors underlying the negative correlation between plasma LPS concentration and age was explored. In a multivariate regression analysis with plasma LPS concentration as the dependent variable and age as the independent variable, sex and BMI, representative demographic variables, were added as covariates, and the effects on the *p*-value were evaluated. The results showed that the statistically significant correlation between plasma LPS concentration and age was maintained even after adjusting for sex (Table 5, Model 2, *p* = 0.003) or BMI (Table 5, Model 3, *p* = 0.002). Therefore, for clinical parameters in NAFLD patients that showed a significant correlation or trend with plasma LPS concentration, univariate correlation analysis between each parameter and age was performed using Spearman’s rank correlation. The results showed that the FIB-4 index (*p* < 0.0001), M2BPGi (*p* = 0.004), hyaluronic acid (*p* = 0.0007), use of ARB (*p* = 0.03), and use of CCB (*p* = 0.004) all showed significant correlations with age. Since the FIB-4 index uses age in its calculation, it was excluded from the analysis. The remaining age-associated parameters were added as covariates in the multivariate regression analysis to evaluate the association between plasma LPS concentration and age. The results showed that even after adding M2BPGi or hyaluronic acid, the significant association between age and plasma LPS concentration was maintained (Table 5, Model 4, *p* = 0.01; and Table 5, Model 5, *p* = 0.03). However, when adjusting for the use of ARB or CCB, the association was substantially attenuated (Table 5, Model 6, *p* = 0.09).

**Figure 5:**
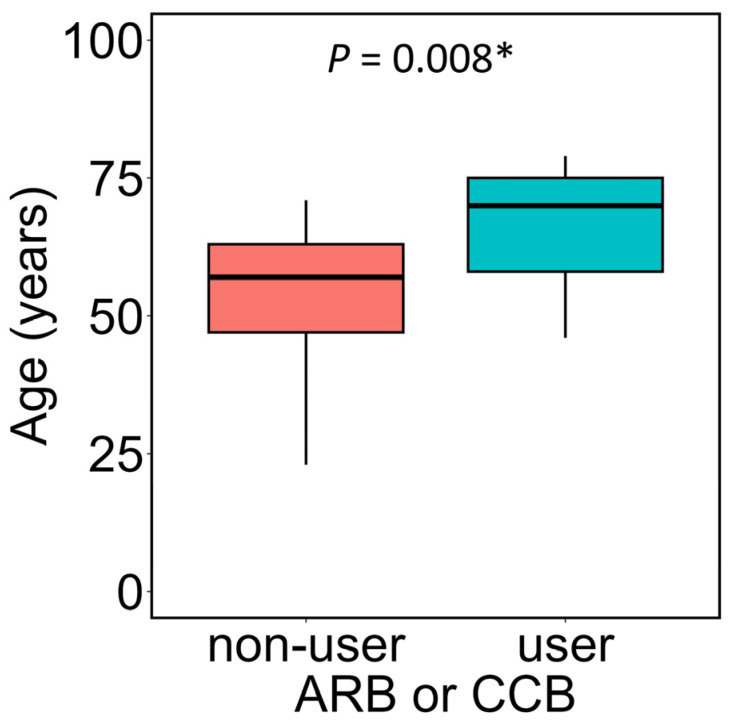
Association between the use of ARB or CCB and age. NAFLD patients were divided into groups of ARB or CCB users and non-users, and a comparison of age between the groups was performed. In the box plot, the horizontal lines represent the first quartile, median, and third quartile, and the ends of the whiskers represent the minimum and maximum values. The group not taking either ARB or CCB, and the users of ARB or CCB, consisted of n = 17 and n = 13, respectively. Mann–Whitney *U* test was used, * *p* < 0.05. ARB, angiotensin II receptor blocker; CCB, calcium channel blocker.

Therefore, the relationship between the use of ARB or CCB and age was examined in NAFLD patients. When comparing the age of users of ARB or CCB with that of non-users, the age of users was significantly higher (Figure 5, *p* = 0.008). Based on these results, it was considered that the use of antihypertensive medications, such as ARB or CCB, may partly contribute to the negative correlation between plasma LPS concentrations and age observed in NAFLD patients.

Therefore, the factors responsible for the negative correlation between the use of ARB or CCB and plasma LPS concentrations in NAFLD patients were explored to assess potential confounding effects. Multivariate regression analysis was performed with plasma LPS concentration as the dependent variable, the use of ARB or CCB as the independent variable, and sex, BMI, blood pressure (SBP, DBP), lipid metabolism indices (LDL-c, HDL-c, TG, TC), and liver function indices (ALT, AST, γ-GT) as covariates. In performing the multivariate regression analysis, the normality of each variable was assessed using the Shapiro–Wilk test. As a result, SBP (*p* = 0.02), ALT (*p* = 0.008), and γ-GT (*p* = 1 × 10^−5^) were found to be non-normally distributed, and their log-transformed values were therefore used in the analysis. The results showed that the significant association between plasma LPS concentrations and the use of ARB or CCB was maintained across all models (Table 6). Based on these results, it was concluded that the use of ARB or CCB affects plasma LPS concentrations independently of blood pressure, and that this influence is independent of both LPS detection sensitivity and its clearance from the bloodstream (for a detailed discussion, refer to Section 4).

### 3.5. Associations Between Plasma LBP Concentrations and Clinical Parameters in All Study Participants, Healthy Individuals, and NAFLD Patients

**Figure 6:**
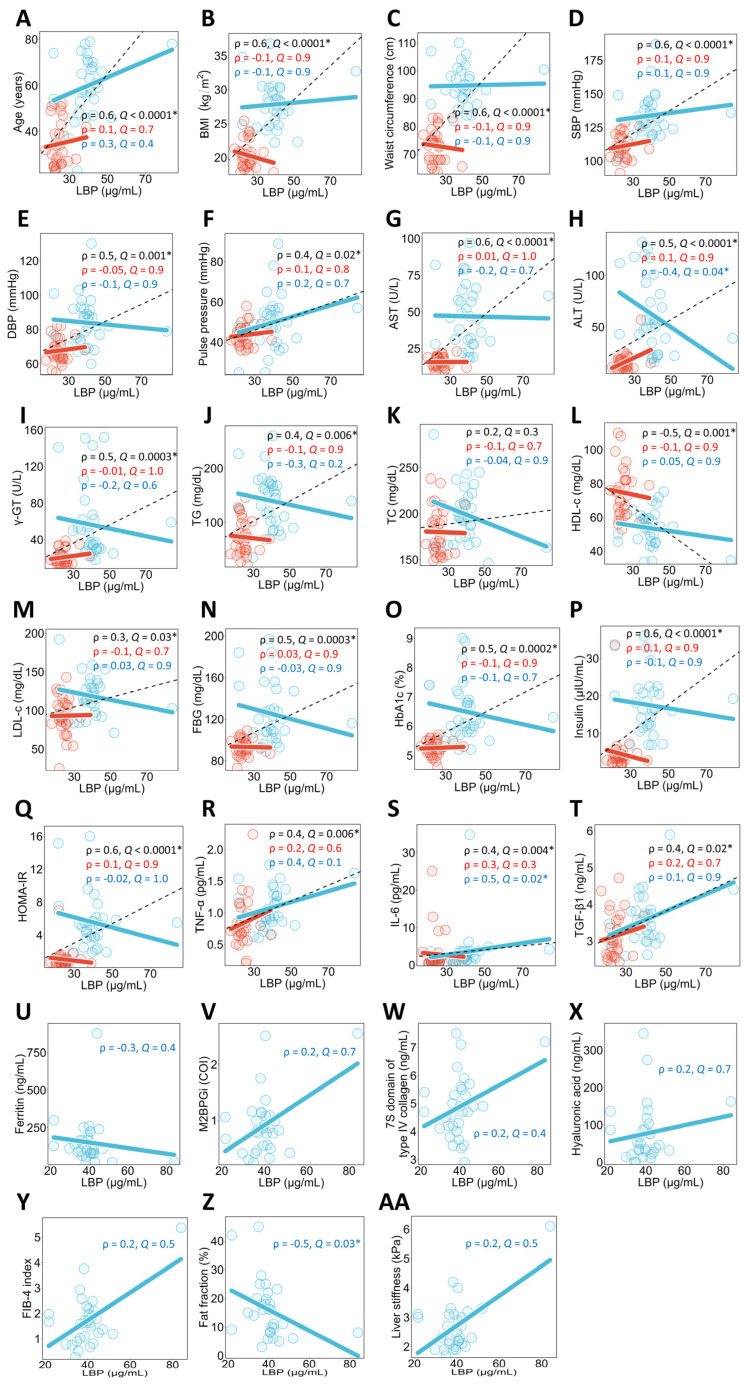
Univariate correlation analysis of plasma LBP concentrations and clinical parameters. Univariate correlation analyses between plasma LBP concentrations and each clinical parameter were conducted using Spearman’s rank correlation in all study participants, healthy individuals, and NAFLD patients. Sub-figures are grouped by clinical categories: (**A**) age; (**B**,**C**) obesity-related indices; (**D**,**E**) blood pressure; (**F**) pulse pressure; (**G**–**I**) liver function markers; (**J**–**M**) lipid metabolism markers; (**N**–**Q**) glucose metabolism markers; (**R**–**T**) inflammatory markers; (**U**–**AA**) NAFLD-related markers. The black dashed line in the graph represents the regression line for the correlation between plasma LBP concentrations and each clinical parameter in all study participants, the red solid line in healthy individuals, and the blue solid line in NAFLD patients. The red and blue circles represent the individual values for healthy individuals and NAFLD patients, respectively. The black, red, and blue text in the graph show the Spearman’s rank correlation coefficient (ρ) and *Q*-value for all study participants, healthy individuals, and NAFLD patients. The sample sizes for all study participants, healthy individuals, and NAFLD patients were n = 62, n = 31, and n = 31, respectively. * *Q* < 0.05. NAFLD, non-alcoholic fatty liver disease; BMI, body mass index; SBP, systolic blood pressure; DBP, diastolic blood pressure; AST, aspartate aminotransferase; ALT, alanine aminotransferase; γ-GT, γ-glutamyltransferase; TG, triglycerides; TC, total cholesterol; HDL-c, high-density lipoprotein cholesterol; LDL-c, low-density lipoprotein cholesterol; FBG, fasting blood glucose; HbA1c, hemoglobin A1c; HOMA-IR, homeostasis model assessment of insulin resistance; TNF-α, tumor necrosis factor-α; IL-6, interleukin-6; TGF-β1, transforming growth factor-β1; LBP, lipopolysaccharide-binding protein; M2BPGi, Mac2 binding protein glycosylation isomer; COI, cut-off index; FIB-4 index, fibrosis-4 index.

A univariate correlation analysis between plasma LBP concentrations and each clinical parameter was conducted for all study participants (Figure 6). The results showed that plasma LBP concentrations were significantly positively correlated with age (Figure 6A), BMI (Figure 6B), waist circumference (Figure 6C), SBP (Figure 6D), DBP (Figure 6E), pulse pressure (Figure 6F), AST (Figure 6G), ALT (Figure 6H), γ-GT (Figure 6I), TG (Figure 6J), LDL-c (Figure 6M), FBG (Figure 6N), HbA1c (Figure 6O), insulin (Figure 6P), HOMA-IR (Figure 6Q), TNF-α (Figure 6R), IL-6 (Figure 6S), and TGF-β1 (Figure 6T), whereas a significant negative correlation was observed with HDL-c (Figure 6L).

Furthermore, univariate correlation analyses between plasma LBP concentrations and clinical parameters were conducted within each group of healthy individuals and NAFLD patients. The results showed that in healthy individuals, there were no clinical parameters significantly correlated with plasma LBP concentrations. In contrast, in NAFLD patients, plasma LBP concentrations were significantly positively correlated with IL-6 (Figure 6S), and significantly negatively correlated with ALT (Figure 6H) and fat fraction (Figure 6Z).

**Figure 7:**
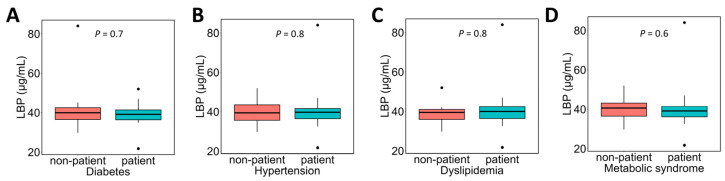
Association between complications and plasma LBP concentrations in NAFLD patients. NAFLD patients were divided into two groups based on the presence or absence of each complication, and a comparison of plasma LBP concentrations between the groups was performed. Subfigures show comparisons according to the presence or absence of the following conditions: (**A**) diabetes, (**B**) hypertension, (**C**) dyslipidemia, and (**D**) metabolic syndrome. In the box plot, the horizontal lines represent the first quartile, median, and third quartile from the bottom, and the ends of the whiskers represent the minimum and maximum values. Dots above or below the maximum or minimum values indicate outliers. The sample sizes (n) for each comparison based on the presence or absence of each condition (yes:no) were as follows: diabetes, 18:13; hypertension, 20:11; dyslipidemia, 23:18; and metabolic syndrome, 20:11. Mann–Whitney *U* test was used. LBP, lipopolysaccharide-binding protein; NAFLD, non-alcoholic fatty liver disease.

The association between the presence or absence of various complications and plasma LBP concentrations in NAFLD patients was evaluated (Figure 7). No significant associations were observed between plasma LBP concentrations and any of the complications.

**Figure 8:**
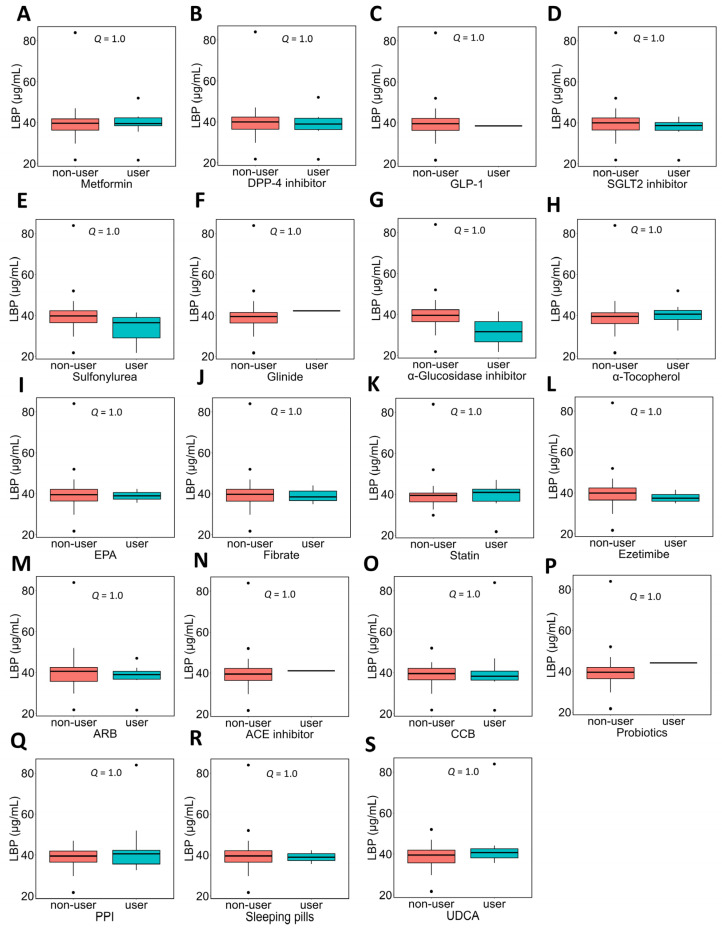
Association between medication use and plasma LBP concentrations in NAFLD patients. NAFLD patients were divided into two groups based on the use of each medication, and a comparison of plasma LBP concentrations between the groups was performed. Subfigures (**A**–**S**) show comparisons of plasma LBP concentrations according to the use or non-use of each medication. In the box plot, the horizontal lines represent the first quartile, median, and third quartile from the bottom, and the ends of the whiskers represent the minimum and maximum values. Dots above or below the maximum or minimum values indicate outliers. The sample sizes (n) for each medication, defined by use or non-use (yes:no), were as follows: metformin, 9:22; DPP-4 inhibitor, 8:23; GLP-1, 1:30; SGLT2 inhibitor, 6:25; sulfonylurea, 3:28; glinide, 1:30; α-glucosidase inhibitor, 2:29; α-tocopherol, 12:19; EPA, 2:29; fibrate, 3:28; statin, 14:17; ezetimibe, 4:27; ARB, 10:21; ACE inhibitor, 1:30; CCB, 9:21; probiotics, 1:30; PPI, 9:22; sleeping pills, 2:29; and UDCA, 8:23. Mann–Whitney *U* test. LBP, lipopolysaccharide-binding protein; NAFLD, non-alcoholic fatty liver disease; DPP4, dipeptidyl peptidase 4; GLP-1, glucagon-like peptide-1; SGLT2, sodium glucose cotransporter 2; EPA, eicosapentaenoic acid; ARB, angiotensin II receptor blocker; ACE, angiotensin-converting enzyme; CCB, calcium channel blocker; PPI, proton pump inhibitor; UDCA, ursodeoxycholic acid.

In NAFLD patients, the association between the use of each medication and plasma LBP concentrations was evaluated (Figure 8). No significant differences in plasma LBP concentrations were observed between users and non-users of any medication.

### 3.6. Multivariate Regression Analysis Between Plasma LBP Concentrations and Clinical Parameters in NAFLD Patients

For the clinical parameters in NAFLD patients that showed a univariate correlation with plasma LBP concentrations, multivariate regression analysis was performed with each clinical parameter as the dependent variable, plasma LBP concentration as the independent variable, and age, sex, and BMI as covariates. In performing the multivariate regression analysis, the normality of each variable was checked using the Shapiro–Wilk test. As a result, plasma LBP concentration (*p* = 5 × 10^−6^), IL-6 (*p* = 2 × 10^−10^), ALT (*p* = 0.008), and fat fraction (*p* = 0.004) were confirmed to be non-normally distributed, and their log-transformed values were therefore used in the multivariate regression analysis. The results showed that for IL-6 and ALT, when no adjustment was made for covariates, a trend of association with plasma LBP concentration was observed (Table 7, Model 1), whereas this trend was not observed after adjustment for age, sex, and BMI (Table 7, Model 2). For fat fraction, a significant negative correlation was observed without adjusting for covariates (Table 7, Model 1), and this trend was maintained even when adjusted for age, sex, and BMI (Table 7, Model 2).

On the other hand, multivariate regression analysis was performed with fat fraction as the dependent variable, plasma LBP concentration as the independent variable, and AST (Model 3), ALT (Model 4), or γ-GT (Model 5) as covariates. In performing the multivariate regression analysis, the normality of AST and γ-GT was confirmed using the Shapiro–Wilk test. As a result, γ-GT was confirmed to be non-normally distributed (*p* = 1 × 10^−5^), and its log-transformed values were therefore used in the multivariate regression analysis. The results showed that even after adding AST and γ-GT as covariates, the significant association or trend between plasma LBP concentrations and fat fraction was maintained (Table 7, Model 3, Model 5). However, when ALT was used as a covariate, the significant correlation with LBP was no longer statistically significant (Table 7, Model 4).

### 3.7. Association Between Plasma LPS Concentrations and Plasma LBP Concentrations

**Figure 9:**
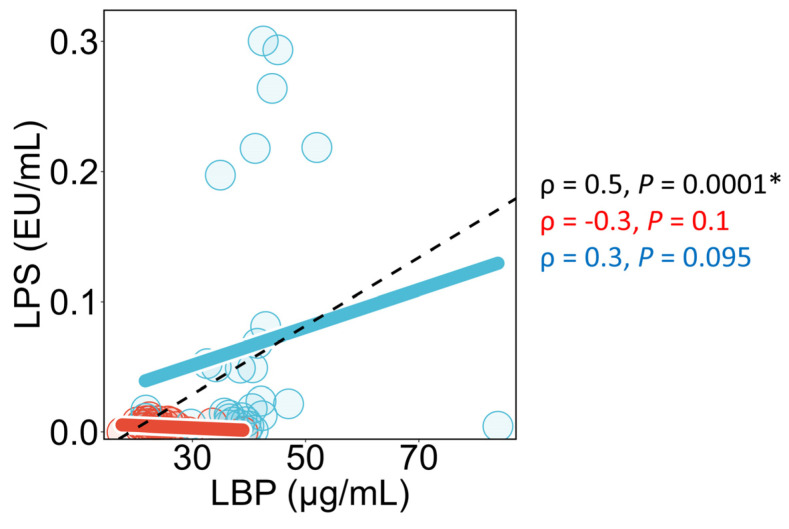
Univariate correlation analysis of plasma LPS concentrations and plasma LBP concentrations. Univariate correlation analyses between plasma LPS concentrations and plasma LBP concentrations were conducted using Spearman’s rank correlation in all study participants, healthy individuals, and NAFLD patients. The black dashed line in the graph represents the regression line for the correlation between plasma LPS concentrations and plasma LBP concentrations in all study participants, the red solid line represents that in healthy individuals, and the blue solid line represents that in NAFLD patients. The red and blue circles represent the individual values for healthy individuals and NAFLD patients, respectively. The black, red, and blue text in the graph show the Spearman’s rank correlation coefficient (ρ) and *p*-value for all study participants, healthy individuals, and NAFLD patients. The sample sizes for all study participants, healthy individuals, and NAFLD patients were n = 62, n = 31, and n = 31, respectively. * *p* < 0.05. LPS, lipopolysaccharide; LBP, lipopolysaccharide-binding protein; NAFLD, non-alcoholic fatty liver disease.

In all study participants, plasma LPS concentrations and plasma LBP concentrations showed significant positive correlation (Figure 9). In healthy individuals, no significant correlation was observed between plasma LPS concentrations and plasma LBP concentrations. On the other hand, in NAFLD patients, a trend toward a positive correlation between these two measurements was observed (*p* = 0.095).

**Figure 10:**
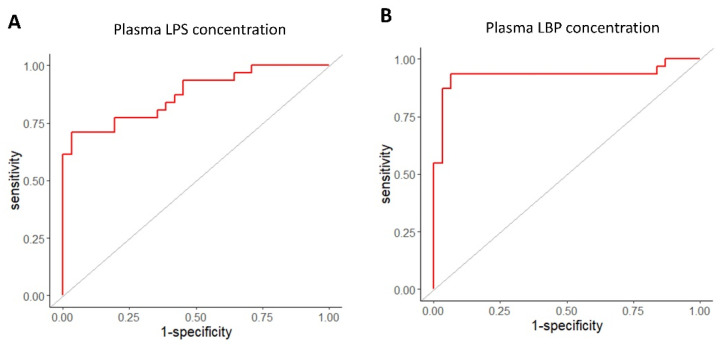
ROC curve for predicting NAFLD. The ROC curves using plasma LPS concentrations (**A**) and plasma LBP concentrations (**B**) are shown. ROC, receiver operating characteristic; LPS, lipopolysaccharide; LBP, lipopolysaccharide-binding protein; NAFLD, non-alcoholic fatty liver disease.

The ability of plasma LPS concentrations and plasma LBP concentrations to discriminate NAFLD was evaluated (Figure 10). For plasma LPS concentrations, the area under the ROC curve (AUC) was 0.87, and the optimal cut-off value defined as the point closest to the upper-left corner of the ROC curve, was 0.010 EU/mL, with a sensitivity of 71% and specificity of 97%. On the other hand, for plasma LBP concentrations, the area under the ROC curve (AUC) was 0.93, and the optimal cut-off value was 29.7 µg/mL, with a sensitivity of 94% and specificity of 94%.

## 4. Discussion

To date, it has remained unclear how exposure to plasma LPS correlates with health status during the gradual deterioration of energy metabolism from a healthy state to the onset and progression of NAFLD. Accordingly, we evaluated the associations between various clinical parameters and plasma LPS and LBP concentrations separately in healthy individuals and patients with NAFLD.

Because proposed mechanisms linking LPS exposure to metabolic dysfunction involve inflammatory pathways, it is important to first confirm whether inflammatory activity is associated with metabolic abnormalities in the present study population. TNF-α has been implicated in the development and progression of NAFLD through inflammation-mediated insulin resistance and fibrotic processes [28,29]. Similarly, IL-6 has been reported to contribute to blood pressure elevation by interacting with the angiotensin II pathway and promoting oxidative stress, endothelial dysfunction, and vascular remodeling [30]. In the present study, positive associations of TNF-α with insulin-related and fibrosis-related markers, and of IL-6 with blood pressure, were observed in NAFLD patients. These findings are consistent with previous reports and indicate that the present study population was suitable for evaluating, at least in part, the relationship between inflammation and metabolic dysfunction.

### 4.1. Plasma LPS Concentration

In this study, consistent with previous reports [13], plasma LPS concentrations in NAFLD patients were higher than those in healthy individuals. Additionally, in the univariate regression analysis for all study participants, plasma LPS concentrations were correlated with indicators of obesity, blood pressure, liver function, lipid metabolism, and glucose metabolism. These findings are consistent with previous reports [12,31,32].

In healthy individuals, plasma LPS concentrations showed significant positive correlations with SBP, γ-GT, and TG. Among these, TG was independently associated with plasma LPS concentrations, even in healthy individuals with no abnormal clinical test values, after adjustment for age, sex, and BMI. Previous studies administering exogenous LPS to healthy individuals have reported an increase in TG levels following LPS administration [33]. However, there have been no reports indicating that endogenous plasma LPS concentrations correlate with TG in healthy individuals. Fasting blood TG are produced when fatty acids released from adipose tissue are absorbed by the liver, where they are synthesized into TG. These TG molecules are then incorporated into very low-density lipoproteins (VLDL) and secreted into the bloodstream [34]. During this process, a proportion of hepatic fatty acids undergo β-oxidation [34]. Two mechanisms by which LPS may affect TG synthesis and metabolism have been proposed. The first mechanism is that LPS promotes de novo TG synthesis in the liver [35]. However, it has been reported that most TG synthesized *de novo* in the liver is not incorporated into VLDL [36]. Additionally, if LPS induced hepatic TG accumulation in the liver, a correlation between plasma LPS concentrations and liver function markers would be expected in healthy individuals with no abnormal clinical test values. However, no such association was observed in this study. Therefore, despite the strong correlation between plasma LPS concentrations and TG in healthy individuals, it is suggested that ectopic TG induction in the liver may not be related. The second mechanism involves the inhibitory effect of LPS on adipocyte browning. Browning adipocytes, also referred to as brown or beige adipocytes, are characterized by a high content of mitochondria. In these cells, free fatty acids are generated from TG, and energy is produced through the mitochondrial respiratory chain via the formation of a proton gradient, resulting in adenosine triphosphate (ATP) synthesis. In addition, these adipocytes can dissipate energy as heat through proton influx into mitochondria mediated by uncoupling protein 1 (UCP1). Due to these characteristics, brown adipocytes are considered major consumers of TG. In fact, activation of brown adipose tissue has been reported to be associated with a decrease in TG levels [37]. It is also suggested that LPS inhibits adipocyte browning. Specifically, LPS activates TLR4 on adipocytes, inducing endoplasmic reticulum stress and autophagy, which leads to mitochondrial dysfunction and suppression of UCP1 expression [38]. Additionally, it has been reported that inflammatory cytokines produced by macrophages under LBP or LPS stimulation suppress adipocyte browning [39,40]. In this study, no significant correlation was observed between plasma LBP concentrations and TG in healthy individuals. Furthermore, the association between plasma LPS concentrations and TG in healthy individuals was appeared to be independent of inflammatory cytokine levels. Therefore, the observed correlation between plasma LPS concentrations and TG in healthy individuals may reflect direct stimulation of adipocytes by LPS and subsequent inhibition of adipocyte browning. If the association between plasma LPS concentrations and TG levels observed in this study reflects suppression of adipocyte browning, our findings could offer valuable insights for preventive medicine. As described in the Introduction, excessive circulating lipids contribute to the development of insulin resistance in both skeletal muscle and adipose tissue during the early stages of NAFLD. In this context, impaired function of brown and beige adipocytes—which play a critical role in lipid clearance—has been suggested as a risk factor for the future development of NAFLD. This concept is supported by evidence from both murine [41] and human [42] studies, which indicate that reduced thermogenic adipocyte activity is associated with dysregulated lipid metabolism and increased susceptibility to NAFLD. However, given the cross-sectional nature of this study, this association should be interpreted as a stage-specific relationship within healthy individuals, rather than as evidence of a temporal or causal link between LPS exposure and alterations in lipid metabolism. As described in the Introduction, abnormalities in carbohydrate metabolism in NAFLD are primarily characterized by progressive hepatic insulin resistance and enhanced gluconeogenesis, both of which contribute to elevated blood glucose levels as the disease advances. In the earlier stages, increased insulin resistance in skeletal muscle and adipose tissue is thought to precede overt hyperglycemia, leading to elevated circulating insulin levels and increased HOMA-IR. It has also been reported that lipopolysaccharide can induce insulin resistance in human skeletal muscle cells in vitro [43]. However, in the present study, no significant associations were found between plasma LPS concentrations and conventional glucose metabolism–related parameters in healthy individuals. This may reflect the limitations of fasting glucose–based indices in capturing early skeletal muscle insulin resistance. Indeed, previous studies using more sensitive approaches, such as the two-step hyperinsulinemic–euglycemic clamp, have demonstrated reduced insulin sensitivity in progression groups even when fasting glucose levels remain unchanged [44]. Taken together, these findings suggest that early impairments in carbohydrate metabolism and mitochondrial energy handling, including TCA cycle–related processes, may not have been fully captured by the parameters used in this study.

On the other hand, in NAFLD patients, plasma LPS concentrations showed a significant negative correlation with age and FIB-4. However, the significant association between FIB-4 index and plasma LPS concentrations was attenuated in the multivariate regression analysis, indicating that the inverse association observed in the univariate analysis was largely driven by age-related confounding rather than reflecting a biologically meaningful inverse effect of endotoxemia on fibrosis progression (the associations between plasma LPS concentrations and age, as well as the use of ARB or CCB, are discussed below). Previous cross-sectional studies focusing exclusively on NAFLD patients have observed a positive correlation between plasma LPS concentrations and liver stiffness as well as liver fibrosis stage [45]. On the other hand, some studies including both NAFLD patients and non-NAFLD individuals have reported no association between plasma LPS concentrations and liver stiffness [12]. Therefore, previous studies have yielded inconsistent results regarding the relationship between plasma LPS concentrations and liver stiffness, and further research is needed to accumulate more findings. Additionally, the association between plasma LPS concentrations and TG observed in healthy individuals was not observed in NAFLD patients. Although a ceiling effect or disease-related metabolic saturation could theoretically contribute to the attenuation of correlations in NAFLD patients, our data do not provide direct evidence to support this mechanism. TG concentrations in NAFLD patients showed substantial inter-individual variability (Table 1), suggesting that a simple upper-limit saturation is unlikely to fully explain the absence of a correlation. Rather, in NAFLD patients, abnormalities in lipid metabolism occur, such as increased fatty acid release from adipose tissue related to peripheral insulin resistance, enhanced triglyceride uptake associated with increased CD36 expression in the liver, increased de novo lipogenesis, lipolysis in both intrahepatic and abdominal fat, and excessive production of VLDL particles [46]. Namely, the plasma TG concentrations in NAFLD patients are greatly influenced by different factors compared to healthy individuals, and the impact of plasma LPS concentrations on plasma TG levels may be smaller in NAFLD patients than in healthy individuals.

Here, although it deviates from the main topic of this paper, we discuss the association between antihypertensive medication use and plasma LPS concentrations observed in NAFLD patients. In NAFLD patients, a negative correlation was observed between plasma LPS concentrations and age. The results of the multivariate regression analysis showed that the use of antihypertensive medications (ARB and CCB) was associated with lower plasma LPS concentrations, and since the age of these users was higher, it was considered the negative correlation between plasma LPS concentrations and age was influenced by this factor. The association between plasma LPS concentrations and the use of ARB or CCB remained even when adjusted for blood pressure, lipid metabolism-related markers (LDL, HDL, TG, TC), and liver function markers (ALT, AST, γ-GTP). From this result, it was suggested that the use of ARB and CCB affects plasma LPS concentrations independent of blood pressure, the sensitivity of LPS detection in the blood due to lipoproteins [47] and the clearance of LPS in the liver [48]. Therefore, I would like to discuss the possibility that ARB and CCB directly contributed to the reduction in plasma LPS concentrations. Angiotensin-converting enzyme 2 (ACE2) is an enzyme that converts angiotensin II into angiotensin-(1-7) and is highly expressed in the intestines [49]. Studies using ACE2 knockout mice have shown that the inability to convert angiotensin II results in the accumulation of angiotensin II [50], and that intestinal organoids exhibit increased intracellular calcium concentrations, a reduction in intestinal stem cells, and increased intestinal permeability [51]. It has been suggested that intracellular calcium concentrations impact tissue repair in the gastrointestinal tract [52]. In other words, the increase in cytosolic calcium levels caused by angiotensin II may lead to impaired intestinal barrier repair, and the combination of CCB and ARB may contribute to maintaining the intestinal barrier function by inhibiting this pathway. Additionally, in the colon, L-cells exclusively express angiotensin II receptors, and it has been reported that angiotensin II stimulation promotes peptide YY (PYY) secretion through the increase in intracellular calcium concentrations in L-cells [53]. In PYY knockout mice, the expression of MUC2 and tight junction-related genes in the intestine was found to be elevated [54], suggesting that PYY contributes to the reduction in intestinal barrier function. Therefore, it is also possible that ARB and CCB inhibited the secretion of PYY from colonic L-cells, thereby contributing to the maintenance of the intestinal barrier. The association observed between the use of ARB or CCB and plasma LPS concentrations in this study may lead to the development of methods to reduce plasma LPS concentrations, and further research is anticipated.

### 4.2. Plasma LBP Concentration

In this study, as previously reported [13], plasma LBP concentrations in NAFLD patients were higher than those in healthy individuals. Additionally, in the univariate regression analysis for all study participants, plasma LPS concentrations were correlated with age, obesity, blood pressure, liver function, lipid metabolism markers, glucose metabolism-related markers, and inflammatory cytokines. This finding was consistent with previous reports (age [22], obesity [45], blood pressure [22], liver function [12,55], lipid metabolism markers [12], glucose metabolism markers [12,22,45], and inflammatory cytokines [56])

In healthy individuals, no clinical parameters were significantly associated with plasma LBP concentrations. On the other hand, in NAFLD patients, plasma LBP concentrations were significantly positively correlated with IL-6 and significantly negatively correlated with ALT and fat fraction. Among these, only fat fraction showed a similar trend with plasma LBP concentrations even after adjustment for age, sex, and BMI in the multivariate regression analysis. However, the association between plasma LBP concentrations and fat fraction was attenuated after adjusting for ALT, an indicator of hepatocyte injury. It is known that LBP is produced by hepatocytes in the liver [14]. On the other hand, an increase in fat fraction leads to hepatocyte injury [57]. Therefore, the negative correlation observed between plasma LBP concentrations and fat fraction in NAFLD patients may reflect liver fat accumulation, which causes hepatocyte damage and reduces LBP production. Namely, no clear association between plasma LBP concentrations and health status was observed in either healthy individuals or NAFLD patients.

### 4.3. Hypothesis Regarding the Differences in Results Between Plasma LPS Concentrations and Plasma LBP Concentrations

In this study, a positive correlation was observed between plasma LPS concentrations and TG in healthy individuals, but no such correlation was found with plasma LBP concentrations. To investigate this further, a univariate correlation analysis between plasma LPS concentrations and plasma LBP concentrations was performed. The results showed a significant positive correlation between plasma LPS concentrations and plasma LBP concentrations in all study participants, but no significant correlation was found in healthy individuals. Previous reports have shown that a high-fructose diet for 3 days significantly increases plasma LPS concentrations (with an increase of approximately 0.1 EU/mL) [58]. During this period, the expression of *TLR4* and *MYD88* mRNA in peripheral blood mononuclear cells also significantly increased, suggesting that even this increase in plasma LPS concentration is sufficient to trigger an inflammatory response via TLR4. However, no increase in plasma LBP concentrations was observed during this period. On the other hand, when LPS is administered intravenously at a dose of 2 ng/kg (this dose is likely higher than the physiological LPS concentration observed in this study, as doses of 2 ng/kg have been reported to cause symptoms such as headache and chills), an increase in plasma LBP concentrations has been observed [33]. Therefore, it is suggested that a certain concentration and duration of LPS exposure are required for plasma LBP concentrations to increase. Additionally, LBP is produced by hepatocytes [14] and adipocytes [59], and it has been reported that LBP production can be stimulated by factors other than LPS, such as free fatty acids [60] and IL-22 [14]. Therefore, in a population including individuals with significantly different histories of LPS exposure, such as healthy individuals and NAFLD patients, plasma LPS concentrations and plasma LBP concentrations are correlated. However, in a population with a narrower distribution range of plasma LPS concentrations, such as healthy individuals only, the influence of factors other than LPS on plasma LBP concentrations may act as noise, which could reduce the correlation between plasma LPS concentrations and plasma LBP concentrations.

On the other hand, in terms of the ability to distinguish between healthy individuals and NAFLD patients, plasma LBP concentrations showed higher sensitivity and a higher AUC compared to plasma LPS concentrations. A similar finding has been observed in the relationship between blood glucose levels and HbA1c (an indicator of long-term exposure to hyperglycemia), where the ability to assess mild cognitive impairment in type 2 diabetes patients is reported to be higher for HbA1c than for blood glucose levels [61]. Therefore, when evaluating the relationship between high-dose and long-term exposure and health status, plasma LBP concentrations may be more useful than plasma LPS concentrations.

## 5. Limitations

This study has several limitations. First, it was conducted among residents of Asahikawa City in Hokkaido, Japan. Therefore, further research is needed to determine whether these findings can be extrapolated to other countries, regions, or ethnicities. Second, there may be insufficient control for confounding factors. It has been reported that plasma LPS concentrations are influenced by lifestyle factors, including diet and exercise [62,63]. However, in this study, dietary and exercise habits were not investigated, and it cannot be ruled out that these factors may have influenced the findings. Instead, BMI was included as an adjustment variable in the multivariate regression analyses between plasma LPS concentrations and clinical parameters. BMI has been reported to show inverse associations with healthy dietary patterns and physical activity levels [64], and is commonly used as a proxy indicator of overall energy balance and lifestyle-related factors in epidemiological studies [65]. Therefore, although residual confounding by lifestyle factors may remain, adjustment for BMI may have partially accounted for differences related to healthy lifestyle behaviors. Third, the healthy individuals who participated in this study were hospital staff, and they are likely to differ significantly from the general population in terms of health awareness, infection risk, and access to medical services. These factors may have influenced the results of this study. Fourth, since this study is cross-sectional, it is not possible to infer causality from the correlations observed in this study. Fifth, the sample size in this study was based on clinical parameters that have been reported to strongly correlate with plasma LPS concentrations. Therefore, there is a concern about false negatives in clinical parameters that show weaker correlations with plasma LPS or LBP concentrations. Sixth, glucose metabolism was evaluated using fasting-based indices, which may not adequately capture early skeletal muscle insulin resistance. Therefore, early alterations in carbohydrate metabolism may not have been fully detected in this study. Seventh, although a recent systematic review and meta-analysis has suggested that circulating LPS concentrations are associated with the presence and severity of NAFLD [66], indicating that peripheral plasma LPS may partially capture biologically relevant endotoxin exposure affecting the liver, plasma LPS concentrations measured in peripheral blood do not directly reflect liver-specific endotoxin exposure via the portal circulation. Gut-derived LPS enters the portal vein and is delivered to the liver, where a substantial proportion is cleared or neutralized before reaching the systemic circulation [67]. Therefore, peripheral plasma LPS concentrations represent the net result of intestinal translocation and hepatic clearance rather than a direct measure of hepatic endotoxin exposure. This physiological dissociation between portal and systemic endotoxin levels may partly explain the lack of a clear association between plasma LPS concentrations and indices of NAFLD severity in the present study, and should be considered when interpreting peripheral LPS measurements in relation to liver-specific pathology. Taken together, given the cross-sectional design, potential residual confounding, limitations in the sensitivity of metabolic indices, and the interpretative constraints of peripheral plasma LPS measurements, the findings of the present study should be interpreted with caution. Further large-scale longitudinal studies incorporating detailed lifestyle assessments and more sensitive and precise measurements of metabolic dysfunction and endotoxin exposure will be required to clarify the temporal and causal relationships between endotoxin exposure and metabolic dysfunction.

## 6. Conclusions

This study demonstrated that plasma LPS concentrations were positively correlated with plasma TG in healthy individuals, while no such correlation was detected in NAFLD patients. Similarly, no such correlation was detected with plasma LBP concentrations. Notably, the correlation between plasma LPS concentrations and circulating TG was consistently observed even in healthy individuals who showed no abnormal values in routine blood biochemical tests. Previous studies have suggested that plasma LPS may negatively affect lipid metabolism [68], but it was unclear at which stage in the process from a healthy state to the onset of NAFLD LPS is involved. The findings of this study suggest that plasma LPS, which has entered the bloodstream, may already be influencing lipid metabolism at the stage where individuals are still considered healthy in routine health screenings. Previous studies on the relationship between LPS and NAFLD have primarily focused on NAFLD patients, but the results of this study highlight the need to further investigate the relationship between plasma LPS and health status in healthy individuals as part of preventive strategies for NAFLD. Future research should focus on the dynamics of LPS in healthy individuals and its long-term health effects, particularly on lipid metabolism. This approach is expected to contribute to the development of early preventive strategies for NAFLD and ultimately improve public health.

## Figures and Tables

**Table 1:** Participant characteristics.

Variable	Healthy Individuals	NAFLD Patients	*p*
N	31	31	NA
Age (years)	31 (26–43)	59 (54–70)	<0.0001 *
Sex (male:female)	13:18	13:18	1
Current habitual drinker (yes:no)	5:26	2:29	0.2
Current habitual smoker (yes:no)	1:30	3:28	0.2
BMI (kg/m^2^)	20.1 (18.7–21.1)	27.7 (25.2–29.9)	<0.0001 *
Waist circumference (cm)	74 (68–77)	94 (90–99)	<0.0001 *
SBP (mmHg)	110 (103–119)	132 (124–142)	<0.0001 *
DBP (mmHg)	66 (63–71)	82 (75–92)	<0.0001 *
Pulse pressure (mmHg)	43 (40–47)	50 (42–56)	0.008 *
AST (U/L)	15 (13–19)	46 (29–61)	<0.0001 *
ALT (U/L)	15 (11–19)	54 (37–88)	<0.0001 *
γ-GT (U/L)	18 (15–24)	37 (30–62)	<0.0001 *
TG (mg/dL)	70 (49–97)	145 (103–170)	<0.0001 *
TC (mg/dL)	170 (159–196)	195 (175–215)	0.01 *
HDL-c (mg/dL)	75 (63–82)	54 (46–60)	<0.0001 *
LDL-c (mg/dL)	91 (83–110)	116 (100–133)	0.0002 *
FBG (mg/dL)	92 (88–99)	120 (108–139)	<0.0001 *
HbA1c (%)	5.3 (5.1–5.5)	6.2 (5.8–7)	<0.0001 *
Insulin (μIU/mL)	3.5 (2.4–4.4)	16.4 (12.1–21)	<0.0001 *
HOMA-IR	0.8 (0.5–1.1)	5.2 (3.7–5.9)	<0.0001 *
TNF-*α* (pg/mL)	0.80 (0.66–0.97)	1.03 (0.86–1.28)	0.002 *
IL-6 (pg/mL)	1.09 (0.72–1.77)	2.53 (1.26–3.66)	0.02*
TGF-*β*1 (ng/mL)	3.02 (2.62–3.58)	3.3 (3.12–3.82)	0.005 *
LPS (EU/mL)	0.0038 (0–0.0072)	0.0169 (0.0078–0.06)	<0.0001 *
LBP (μg/mL)	23.9 (22.3–25.8)	39.6 (36.4–42.2)	<0.0001 *
Ferritin (ng/mL)	NA	109 (79–174)	NA
M2BPGi (COI)	NA	0.82 (0.53–1.09)	NA
7S domain of type IV collagen (ng/mL)	NA	4.7 (4.2–5.5)	NA
Hyaluronic acid (ng/mL)	NA	51 (29–96)	NA
FIB-4 index	NA	1.63 (1.06–2.04)	NA
Fat fraction (%)	NA	15.5 (8.5–19.9)	NA
Fat grade (G1:G2:G3:NA)	NA	13:5:12:1	NA
Liver stiffness (kPa)	NA	2.4 (2.1–3.1)	NA
Liver stiffness grade (F0:F1–2:F3–4:NA)	NA	16:11:3:1	NA
Diabetes (yes:no)	NA	18:13	NA
Hypertension (yes:no)	NA	20:11	NA
Dyslipidemia (yes:no)	NA	23:18	NA
Metabolic syndrome (yes:no)	NA	20:11	NA
Medication			
Metformin (yes:no)	NA	9:22	NA
DPP-4 inhibitor (yes:no)	NA	8:23	NA
GLP-1 (yes:no)	NA	1:30	NA
SGLT2 inhibitor (yes:no)	NA	6:25	NA
Sulfonylurea (yes:no)	NA	3:28	NA
Glinide (yes:no)	NA	1:30	NA
*α*-Glucosidase inhibitor (yes:no)	NA	2:29	NA
*α*-Tocopherol (yes:no)	NA	12:19	NA
EPA (yes:no)	NA	2:29	NA
Fibrate (yes:no)	NA	3:28	NA
Statin (yes:no)	NA	14:17	NA
Ezetimibe (yes:no)	NA	4:27	NA
ARB (yes:no)	NA	10:21	NA
ACE inhibitor (yes:no)	NA	1:30	NA
CCB (yes:no:NA)	NA	9:21:1	NA
Probiotics (yes:no)	NA	1:30	NA
PPI (yes:no)	NA	9:22	NA
Sleeping pills (yes:no)	NA	2:29	NA
UDCA (yes:no)	NA	8:23	NA

NAFLD, non-alcoholic fatty liver disease; BMI, body mass index; SBP, systolic blood pressure; DBP, diastolic blood pressure; AST, aspartate aminotransferase; ALT, alanine aminotransferase; γ-GT, γ-glutamyltransferase; TG, triglycerides; TC, total cholesterol; HDL-c, high-density lipoprotein cholesterol; LDL-c, low-density lipoprotein cholesterol; FBG, fasting blood glucose; HbA1c, hemoglobin A1c; HOMA-IR, homeostasis model assessment of insulin resistance; TNF-α, tumor necrosis factor-α; IL-6, interleukin-6; TGF-β1, transforming growth factor-β1; LPS, lipopolysaccharide; LBP, lipopolysaccharide-binding protein; M2BPGi, Mac2 binding protein glycosylation isomer; COI, cut-off index; FIB-4 index, fibrosis-4 index; DPP4, dipeptidyl peptidase 4; GLP-1, glucagon-like peptide-1; SGLT2, sodium glucose cotransporter 2; EPA, eicosapentaenoic acid; ARB, angiotensin II receptor blocker; ACE, angiotensin-converting enzyme; CCB, calcium channel blocker; PPI, proton pump inhibitor; UDCA, ursodeoxycholic acid; NA, Not Applicable. Continuous variables were compared between groups using the Mann–Whitney *U* test. Categorical variables were compared using Fisher’s exact test. Continuous variables are presented as median (IQR). Categorical variables are presented as n. * *p* < 0.05.

**Table 2:** Multivariate regression analysis of clinical parameters and plasma LPS concentrations in healthy individuals ^1^ (only parameters with significant correlations in Figure 1).

Variable	Model 1		Model 2	
	β	*p*	β	*p*
SBP	1275	0.01 *	836	0.05
γ-GT ^2^	43	0.02 *	33	0.07
TG	5024	0.001 *	5201	0.002 *

* *p* < 0.05. ^1^ Multivariate regression analysis was conducted with each clinical parameter as the dependent variable and plasma LPS concentration as the independent variable. Model 1: No adjustment for covariates, Model 2: Adjusted for age (log-transformed), sex, and BMI (log-transformed). ^2^ Log-transformed values. LPS, lipopolysaccharide; BMI, body mass index; SBP, systolic blood pressure; γ-GT, γ-glutamyltransferase; TG, triglycerides.

**Table 3:** Multivariate regression analysis of clinical parameters and plasma LPS concentrations in healthy individuals with no abnormal clinical test values ^1^ (only parameters with significant correlations in Figure 1).

Variable	Model 1		Model 2		Model 3		Model 4		Model 5		Model 6	
	β	*p*	β	*p*	β	*p*	β	*p*	β	*p*	β	*p*
SBP	1518	0.047 *	982	0.22	NA	NA	NA	NA	NA	NA	NA	NA
γ-GT ^2^	35	0.16	38	0.17	NA	NA	NA	NA	NA	NA	NA	NA
TG	5055	0.01 *	5148	0.04 *	5220	0.04 *	5551	0.05	5055	0.05	5380	0.04 *

* *p* < 0.05. ^1^ Multivariate regression analysis was conducted with each clinical parameter as the dependent variable and plasma LPS concentration as the independent variable. Model 1: No adjustment for covariates, Model 2: Adjusted for age, sex, and BMI, Model 3: Adjusted for plasma LBP concentration (log-transformed) in addition to Model 2, Model 4: Adjusted for plasma TNF-α concentration in addition to Model 2, Model 5: Adjusted for plasma IL-6 concentration (log-transformed) in addition to Model 2, Model 6: Adjusted for plasma TGF-β1 concentration (log-transformed) in addition to Model 2. Models 3 and onwards were performed only for TG, which showed a significant correlation with plasma LPS concentration in Model 2. ^2^ Log-transformed values. LPS, lipopolysaccharide; BMI, body mass index; SBP, systolic blood pressure; γ-GT, γ-glutamyltransferase; TG, triglycerides; LBP, lipopolysaccharide-binding protein; TNF-α, tumor necrosis factor-α; IL-6, interleukin-6; TGF-β1, transforming growth factor-β1.

**Table 4:** Multivariate regression analysis of clinical parameters and plasma LPS concentrations in NAFLD patients (only parameters with significant correlations in Figure 1 or Figure 2).

Variable	Model 1		Model 2		Model 3	
	β	*p*	β	*p*	β	*p*
FIB-4 index ^1,2^	−0.17	0.01 *	−0.01	0.9	−0.04	0.4

* *p* < 0.05. ^1^ Multivariate regression analysis was conducted with each clinical parameter as the dependent variable and plasma LPS concentration as the independent variable. ^2^ Log-transformed values. Model 1: No adjustment for covariates, Model 2: Adjusted for age, sex, and BMI, Model 3: Adjusted for the use of ARB or CCB in addition to Model 2. LPS, lipopolysaccharide; NAFLD, non-alcoholic fatty liver disease; FIB-4 index, fibrosis-4 index.

**Table 5:** Multivariate regression analysis of plasma LPS concentrations and age in NAFLD patients ^1^.

Model	β	*p*
1	−0.06	0.002 *
2	−0.06	0.003 *
3	−0.06	0.002 *
4	−0.06	0.01 *
5	−0.06	0.03 *
6	−0.03	0.09

* *p* < 0.05. ^1^ Multivariate regression analysis was conducted with plasma LPS concentration as the dependent variable and age as the independent variable. Model 1: No adjustment for covariates, Model 2: Adjusted for sex, Model 3: Adjusted for BMI, Model 4: Adjusted for M2BPGi (log-transformed), Model 5: Adjusted for hyaluronic acid (log-transformed), Model 6: Adjusted for the use of ARB or CCB. LPS, lipopolysaccharide; NAFLD, non-alcoholic fatty liver disease; BMI, body mass index; M2BPGi, Mac2 binding protein glycosylation isomer; ARB, angiotensin II receptor blocker; CCB, calcium channel blocker.

**Table 6:** Multivariate regression analysis of plasma LPS concentrations and the use of ARB or CCB in NAFLD patients.

Model	β	*p*
1	−1.97	0.0002 *
2	−2.13	0.0002 *
3	−2.10	0.0004 *
4	−1.99	0.0009 *
5	−2.20	0.0001 *
6	−2.13	0.0002 *
7	−2.15	0.0002 *
8	−2.13	0.0002 *
9	−2.13	0.0003 *
10	−2.12	0.0003 *
11	−2.12	0.0003 *

* *p* < 0.05. Multivariate regression analysis was conducted with plasma LPS concentration (log-transformed) as the dependent variable and the use of ARB or CCB as the independent variable. Model 1: No adjustment for covariates, Model 2: Adjusted for sex and BMI, Model 3: Adjusted for SBP (log-transformed) in addition to Model 2, Model 4: Adjusted for DBP in addition to Model 2, Model 5: Adjusted for TG in addition to Model 2, Model 6: Adjusted for TC in addition to Model 2, Model 7: Adjusted for HDL-c in addition to Model 2, Model 8: Adjusted for LDL-c in addition to Model 2, Model 9: Adjusted for AST in addition to Model 2, Model 10: Adjusted for ALT (log-transformed) in addition to Model 2, Model 11: Adjusted for γ-GT (log-transformed) in addition to Model 2. NAFLD, non-alcoholic fatty liver disease; LPS, lipopolysaccharide; ARB, angiotensin II receptor blocker; CCB, calcium channel blocker; BMI, body mass index; SBP, systolic blood pressure; DBP, diastolic blood pressure; TG, triglycerides; TC, total cholesterol; HDL-c, high-density lipoprotein cholesterol; LDL-c, low-density lipoprotein cholesterol; AST, aspartate aminotransferase; ALT, alanine aminotransferase; γ-GT, γ-glutamyltransferase.

**Table 7:** Multivariate regression analysis of clinical parameters and plasma LBP concentrations in NAFLD patients ^1^ (only parameters with significant correlations in Figure 6).

Variable	Model 1		Model 2		Model 3		Model 4		Model 5	
	β	*p*	β	*p*	β	*p*	β	*p*	β	*p*
IL-6	1.3	0.055	1.0	0.124	NA	NA	NA	NA	NA	NA
ALT	−0.9	0.061	−0.7	0.115	NA	NA	NA	NA	NA	NA
fat fraction	−1.0	0.046 *	−0.9	0.066	−0.9	0.048	−0.6	0.2	−0.9	0.06

* *p* < 0.05. ^1^ Multivariate regression analysis was conducted with each clinical parameter as the dependent variable and plasma LBP concentration as the independent variable. Model 1: No adjustment for covariates, Model 2: Adjusted for age, sex, and BMI, Model 3: Adjusted for AST, Model 4: Adjusted for ALT (log-transformed), Model 5: Adjusted for γ-GT (log-transformed). NAFLD, non-alcoholic fatty liver disease; LBP, lipopolysaccharide-binding protein; IL-6, interleukin-6; ALT, alanine aminotransferase; AST, aspartate aminotransferase; γ-GT, γ-glutamyltransferase.

## Data Availability

The data presented in this study is available on request from the corresponding author. The data are not publicly available due to ethical concerns.

## Abbreviations

The following abbreviations are used in this manuscript:

NAFLD	Non-Alcoholic Fatty Liver Disease
NASH	Non-Alcoholic Steatohepatitis
LPS	Lipopolysaccharide
LBP	Lipopolysaccharide-Binding Protein
TLR4	Toll-Like Receptor 4
TNF-α	Tumor Necrosis Factor-Alpha
IL-6	Interleukin-6
TGF-β1	Transforming Growth Factor-Beta 1
UCP1	Uncoupling Protein 1
PYY	Peptide YY
BMI	Body Mass Index
SBP	Systolic Blood Pressure
DBP	Diastolic Blood Pressure
AST	Aspartate Aminotransferase
ALT	Alanine Aminotransferase
γ-GT	Gamma-Glutamyl Transferase
TG	Triglycerides
TC	Total Cholesterol
HDL-c	High-Density Lipoprotein Cholesterol
LDL-c	Low-Density Lipoprotein Cholesterol
FBG	Fasting Blood Glucose
HbA1c	Hemoglobin A1c
HOMA-IR	Homeostasis Model Assessment of Insulin Resistance
M2BPGi	Mac-2 Binding Protein Glycosylation Isomer
FIB-4	Fibrosis-4 Index
MRE	Magnetic Resonance Elastography
MRI-PDFF	Magnetic Resonance Imaging Proton Density Fat Fraction
ROC	Receiver Operating Characteristic
AUC	Area Under the Curve
IQR	Interquartile Range
FDR	False Discovery Rate
ARB	Angiotensin II Receptor Blocker
ACE	Angiotensin-Converting Enzyme
CCB	Calcium Channel Blocker
DPP-4	Dipeptidyl Peptidase 4
GLP-1	Glucagon-Like Peptide-1
SGLT2	Sodium Glucose Cotransporter 2
EPA	Eicosapentaenoic Acid
PPI	Proton Pump Inhibitor
UDCA	Ursodeoxycholic Acid
LAL	Limulus Amoebocyte Lysate
Abs	Absorbance
ATP	Adenosine Triphosphates
